# A latent profile analysis on adolescents' Non-Suicidal Self-Injury related to intrapersonal and interpersonal factors

**DOI:** 10.1186/s13034-024-00801-4

**Published:** 2024-09-17

**Authors:** Jong-Sun Lee, Sojung Kim, Ji-Hyun Lee, Jae-Won Kim, Jae Hyun Yoo, Doug Hyun Han, Hyunchan Hwang, Chi-Hyun Choi, Dong-Gi Seo

**Affiliations:** 1 Department of Psychology, Kangwon National University, Chuncheon, Kangwon-Do South Korea; 2 Department of Psychology, Yeungnam University, Gyeongsan, Gyeongbuk South Korea; 3 Department of Psychology, College of Social Science, Hallym Applied Psychology Institute, Hallym University, Chuncheon, Kangwon-Do South Korea; 4 Department of Psychiatry & Behavioral Sciences, Division of Child and Adolescent Psychiatry, Seoul National University College of Medicine, Seoul National University Hospital, Seoul National University Children’s Hospital, Seoul, South Korea; 5 Department of Psychiatry, The Catholic University of Korea Seoul St. Mary’s Hospital, Seoul, South Korea; 6 Department of Psychiatry, Chung-Ang University College of Medicine, 84 Heukseok-Ro, Dongjak-Gu, Seoul, 06973 Republic of Korea; 7 Seoul Alpha Neuropsychiatric Clinic, 511 Nonhyeon-Ro, Gangnam-Gu, Seoul, 06131 Republic of Korea

**Keywords:** Adolescent, NSSI, Latent profile analysis, LPA, Self-injury

## Abstract

Non-suicidal self-injury (NSSI) among adolescents continues to be a significant public health concern worldwide. A recent systematic review and meta-analysis found that the global prevalence of NSSI in adolescents aged 12–18 years was 17.2%, with higher rates reported among females (19.7%) than males (14.8%). This behavior has been linked to several negative outcomes, such as depression, anxiety, substance abuse, and suicidal ideation. The present study aimed to classify adolescents based on intrapersonal and interpersonal factors associated with NSSI proposed in Nock’s (2009) integrated model of NSSI, to identify distinct clusters targeting specific risk factors. This encompassed negative cognition, emotional vulnerability, poor coping skill, peer-victimization, family adaptability, and perceived stress. A total of 881 adolescents aged 11–16 years in South Korea completed self-reported questionnaires on automatic thoughts, depression, emotional regulation, peer victimization, family adaptability and perceived stress. Latent profile analysis (LPA) revealed three distinct classes: “the severe group”, “the moderate group”, “the mild group”. Class 3 (“severe group”: *N* = *127*) exhibited greater severity related to NSSI, including negative cognition, emotional vulnerability, poor coping skills, peer victimization, and perceived stress, with weaker levels of factors that can prevent NSSI compared to class 1 (“mild group”: *N* = *416*) and class 2 (“moderated group”: *N* = 338). The present study emphasizes the importance of considering both intrapersonal (e.g., negative automatic thoughts & emotional dysregulation) and interpersonal factors (i.e., peer victimization) when understanding NSSI - among adolescents. These findings can be utilized to develop interventions aimed at reducing the prevalence and severity of NSSI among adolescents.

**Supplementary Information:**

The online version contains supplementary material available at 10.1186/s13034-024-00801-4.

## Introduction

Non-suicidal self-injury (NSSI) refers to deliberate acts of self-harm that do not involve an intention to die [36]. The prevalence of NSSI among adolescents is a growing public health concern worldwide [18]. A meta-analysis of community-based studies conducted between 1990 and 2015 reported an overall lifetime prevalence of NSSI in adolescents of 16.9% [18]. Other meta-studies have estimated the overall lifetime prevalence of NSSI in children and adolescents to be 22.1%, with reported prevalence rates ranging from 2.9% to 69.6% [31]. Given the high comorbidity of NSSI with psychiatric disorders and its significant predictive value for future NSSI [22], early detection and intervention are of utmost importance.

The integrated theoretical model of NSSI by [35] identifies two distinct risk factors contributing to the onset and maintenance of NSSI: intrapersonal and interpersonal factors. Intrapersonal factors primarily focus on individual characteristics such as self-esteem and self-efficacy [47] and include cognitive and emotional vulnerability, particularly in response to stress [17]. These factors play a critical role in regulating emotions, often involving the release of negative emotions, unpleasant tension or psychological pressures [30, 37]. Cha et al. [7] identified negative thought content, such as automatic negative thinking, and negative information processing biases, such as rumination [12], as significant intrapersonal risk factors for self-harm. As emotional vulnerability factors, anger, depression, anxiety, emotion regulation were identified as risk factors related to the onset and maintenance of NSSI in adolescents [4, 5, 9, 10, 16, 43, 51]. On the other hand, interpersonal factors center around interactions with others and serve functions such as seeking attention/support, establishing peer bonds, and experiencing interpersonal influence [17]. Longitudinal studies conducted with college students and admitted adolescent inpatients have demonstrated that engagement in and persistence of NSSI are more closely related to intrapersonal factors [27, 55]. Specifically, Kieken and colleagues (2017) found that higher frequency and multiple instances of NSSI, academic and emotional stress, perceived lack of emotional regulatory capability differentiated between individuals who continued and ceased NSSI in a three-year longitudinal study. In a six-month follow-up study, Yen and colleagues (2016) found that automatic positive reinforcement (APR) and ongoing chronic depression over six months were pivotal reasons for persisting NSSI. However, based on these findings, the authors suggested that individuals who engaging in NSSI for APR might be a group that experiences restricted affect, such as feelings of emptiness, numbness, anhedonia, and detachment [37]. Conversely, substance use was found to increase the high risk of engaging in NSSI but was helpful in reducing the risk of NSSI persistence. These two studies underscore the significant role of intrapersonal factors in the causation and maintenance of NSSI. However, during adolescence, a period characterized by complex social and relational challenges, both intrapersonal and interpersonal factors may be significantly associated with NSSI. Indeed, one longitudinal study investigating the relationships between intrapersonal and interpersonal factors in predicting onset and cessation of NSSI in adolescents found that self-esteem, self-efficacy, and cognitive reappraisal partially mediated the relationship between insecure attachment and NSSI [47]. This study also reported that family support was the strongest predictor for the cessation of NSSI, while lack of support plays a pivotal role in the onset of NSSI, stressing that both intrapersonal (i.e., cognitive restricting) and interpersonal factors (i.e., family support) should be targeted as the main strategies in preventing NSSI in adolescents. Muehlenkamp et al. [32] found that interpersonal functions are more commonly endorsed as the initial reasons for non-suicidal self-harm, which typically occurs during adolescence. In contrast, intrapersonal functions are more likely to underpin self-reported repeated non-suicidal self-harm behaviors. Recognizing the simultaneous impact of these two sets of factors is crucial for comprehending the underlying mechanisms of non-suicidal self-harm behaviors, especially for adolescence. By acknowledging the specific contributions of intrapersonal and interpersonal factors, interventions and prevention strategies can be tailored to address the unique needs of individuals and provide effective support for those at risk.

Non-suicidal self-injury (NSSI) among adolescents is a multifaceted and concerning issue that requires a comprehensive understanding. To address this, it is important to identify subgroups of adolescents engaging in NSSI and explore the distinctive features associated with each group. This can be achieved by a statistical analysis using latent class analysis (LCA) or latent profile analysis (LPA). However, there is a handful of studies on NSSI specifically within adolescent population. Few studies have investigated the psychosocial factors and psychiatric symptoms associated with non-suicidal self-harm behaviors in adolescents. For instance, Yang et al. [54] categorized four groups based on unhealthy psychological factors, including tobacco and alcohol use, unhealthy weight loss, screen time, and mobile phone dependence. They found that students classified in the high-risk cluster were more prone to engaging in multiple unhealthy behaviors, such as unhealthy weight loss, tobacco use, alcohol consumption, excessive screen time, and dependence on mobile phones. Another study by Halladay et al. [21] investigated psychiatric symptoms, including substance use, generalized anxiety disorder (GAD), depression, social phobia (SP), and conduct disorder (CD), among outpatient adolescents aged 12 to 17 years in Canada. This study classified participants into three classes; low substance use with lower frequency and/or severity emotional and behavioral disorder symptoms (class 1), low substance use with higher emotional and behavioral disorder symptoms (class 2), and high in both (class 3). Class 3 differed from the other two classes (class1 & 2) in terms of trauma histories, suicide attempts and aggression, highlighting the need for a multidisciplinary approach for adolescents with complex psychiatric presentations. In a study with first-year undergraduate students, three groups were identified, “healthy group for NSSI and suicidal behavior”, “high risk for NSSI/not high risk for suicidal behavior,” and the “high risk group for NSSI and suicidal behavior.” The results indicated that the high risk group for NSSI and suicidal behavior, considered the most at-risk group, met the clinical cut-off score for suicidal factors such as suicidal attempt, the risk for future suicidal behavior. Furthermore, the group at risk for both NSSI and suicidal behavior significantly differed in psychosocial factors, including daily hassles, difficulties with emotion regulation, depressive symptoms, self-esteem, social anxiety, behavioral inhibition, friendship quality, parental attachment, parental criticism, and parental psychological control [23]. Recent research has categorized adolescent psychiatric patients in Finland into two groups based on indicators of deliberate self-harm frequency and emotional dysregulation [14], while justice-involved adolescents in Hungary have been classified into three groups based on different forms of self-harm behaviors [44]. A recent longitudinal cohort study identified subgroups of NSSI among first-year secondary school adolescents in the Netherlands. This study identified four groups ("Low NSSI–Low suicidality," "Moderate NSSI-Low suicidality," "Moderate NSSI-High suicidality," and "High NSSI-High suicidality") based on the frequency of NSSI and suicidality. In a follow-up analysis, class 4 ("High NSSI-High suicidality") exhibited more internalizing and externalizing problems, less support in relationships, and the lowest levels of self-esteem [13]. However, when these studies identified subgroups of NSSI, class indicators were solely based on the method and frequency of NSSI and suicidal behavior or psychiatric disorders. None of these studies included both intrapersonal and interpersonal factors that Nock [35] indicated as significant factors accounting for NSSI in the integrated theoretical model. According to Nock’s [35] model, understanding both intrapersonal and interpersonal factors is crucial because they offer a comprehensive view of the mechanisms underlying NSSI. Intrapersonal factors such as negative cognition and emotional vulnerability interact with interpersonal factors like peer victimization to contribute to the onset and maintenance of NSSI. For instance, peer victimization or lack of family support might predispose adolescents to start NSSI, while negative cognition and poor self-image can exacerbate and maintain these behaviors. Conversely, adolescents might initially engage in NSSI due to intrapersonal issues, which are then reinforced by negative interpersonal experiences. Including both sets of factors can provide a more nuanced understanding of NSSI, which is helpful for developing effective prevention and intervention strategies in the future. This dual focus is both theoretically sound and practically relevant, as it acknowledges the complex interplay of individual dynamics in the development and persistence of NSSI.

As such, the main aim of the current study is to investigate, for the first time, how intrapersonal and interpersonal factors accounting for NSSI proposed in the integrated model of NSSI by Nock [35] cluster together in NSSI adolescents. To this end, the present study aims to conduct latent profile analysis (LPA) utilizing five domain indicators such as negative cognition, emotional vulnerability, poor coping skills, peer victimization, family adaptability, and perceived stress. Our findings would be the first to aid in understanding the complex dynamic underlying NSSI suggested by the integrated theoretical model by Nock [35]. Clinically and practically, this approach contributes to the development of individually tailored interventions; identifying subgroups based on the class indicators such as cognition, emotion, coping skills, peer and family relationships help determine specific therapeutic target domains for adolescents with NSSI.

## Methods

### Participants

The present study employed a nationwide online survey conducted in South Korea to gather data. Participants were selected from six stratified districts in South Korea, including Seoul, Kyeonggi, Kangwon, Gyeongsang, among others, based on their estimated proportions in the national population. A total of 881 adolescents and their parents voluntarily provided online consent to participate in the study. Inclusion criteria required participants to have reported non-suicidal self-injury (NSSI) at least once in the past 12 months and be in grades six to nine. Once the inclusion criteria were met, the adolescents were invited to complete a series of self-reported questionnaires via a survey webpage accessed through a provided URL. The sample comprised 227 (25.8%) 6th-grade students, 221 (25.85%) 7th-grade, 226 (25.7%) 8th-grade, and 207 (23.5%) 9th-grade students. Among the participants, 487 (55.3%) identified as male, while 394 (44.7%) identified as female. The overall mean age of the participants was 13.91 years (SD = 0.81), with ages ranging from 11 to 16 years. The study received approval from the Institutional Review Board (IRB) of Seoul National University Hospital (IRB Number H- 1904–093-1027) and Kangwon National University (IRB Number KWNUIRB-2020–01-012) to ensure ethical compliance and participant protection. Demographics information is described in Appendix A.

### Measures

#### Center for Epidemiological Studies Depression Scale for Children (CES-DC)

The Center for Epidemiological Studies Depression Scale for Children (CES-DC) was developed by Weissman to assess levels of depression among children and adolescents [50]. CES-DC consists of 20 items with possible scores ranging from 0 to 60. Each item uses a 4-point Likert scale (0–3). Higher CES-DC scores indicate increasing levels of depression. The internal consistency of the scale was 0.936 in this study. Of the 20 items, ten (2, 5, 6, 10, 11, 15, 17, 18, 19, & 20) with high factor loadings were selected and adapted for the depression concept.

#### Automatic Thoughts Questionnaire (ATQ-N)

Automatic Thoughts Questionnaire (ATQ-N) was developed by Hollon and Kendall [25] to measure automatic negative thoughts. ATQ-N consists of 30 items with possible scores ranging from 30 to 150. Each item uses a 5-point Likert scale (1–5). High ATQ-N scores indicate a high tendency to think negatively. In this study, we used the Korean version of the ATQ-N translated and validated by [53]. The internal consistency of the scale was 0.981 in this study. Among the 30 items, 14 items with the low item-total correlation and low factor loading below 0.3, as well as high cross-loading between factors, were removed from the scale [20]. Finally, 16 items (1, 2, 4, 10, 11, 17, 18, 19, 20, 21, 22, 25, 27, 28, 29, and  30) were selected and utilized to measure the concept of negative thinking.

#### Ruminative Response Scale (RRS)

The ruminative Response Scale (RRS) was developed to evaluate ruminative response by Nolen-Hoeksema and Morrow [38]. The Korean version of the Ruminative Response Scale (K-RRS) was validated by Kim et al. [28]. It consists of three subscales (reflection, brooding and depressive rumination) and 22 items with possible scores ranging from 22 to 88. Each item describes the level of thoughts or actions using a 4-point Likert scale (1–4). A higher K-RRS score indicates a higher tendency for ruminative response. The internal consistency of the scale was 0.966 in this study. We used ten items (1, 2, 3, 5, 8, 9, 10, 15, 19, and 22) out of the 22 items. Four items (5, 9, 10, 15) were used for brooding factor, while the other 6 (1, 2, 3, 8, 19, and 22) were used for depressive rumination concept.

#### Difficulties in Emotion Regulation Scale– 16 item version (DERS-16)

DERS-16 is developed by Bjureberg et al. [3] as a brief version of DERS [19]. This brief scale consists of five subscales (lack of emotional clarity, difficulties engaging in goal-directed behavior, impulse control difficulties, limited access to effective emotion regulation strategies, and nonacceptance of emotional response) and a total of 16 items with possible ranging from 16–80. Each item describes difficulties in emotion regulation using a 5-point Likert scale (1–5). A higher DERS-16 score indicates greater difficulties in emotion regulation [3]. The internal consistency of the scale was 0.973 in this study. Thirteen items (1, 2, 3, 4, 5, 8, 9, 11, 12, 13, 14, 15, and 16) were selected and adapted for the difficulties in emotion regulation concept.

#### Toronto Alexithymia Scale (TAS-20)

TAS-20 was developed by Bagby to measure a participant’s inability to identify and describe their own emotions. TAS-20 includes three subscales (difficulty identifying feelings, difficulty describing feelings, and externally oriented thinking). Each item is scored on a 5-point (1–5), Likert-type scale and five reverse-scored items. Total scores ranged from 20 to 100, with higher scores indicating greater impairment [2]. We used TAS-20 K, a Korean version of the TAS-20 developed by Chung et al. [8]. The internal consistency of the scale was 0.900 in this study. Four items (1, 2, 6, and 8) were selected and used for the alexithymia concept.

#### The short form of the Buss-Perry Aggression Questionnaire (BPAQ-SF)

BPAQ-SF was a short form of Buss and Perry’s aggression questionnaire (BPAQ). This short-form scale was developed by Bryant and Smith [6] to measure aggression. BPAQ-SF includes four subscales (physical aggression, verbal aggression, anger, and hostility). A total of 12 items with possible ranging from 12 to 60. Each item uses a 5-point Likert scale (1–5). Higher BPAQ-SF scores indicate higher aggression. The internal consistency of the scale was 0.922 in this study. We used 7 items (2, 3, 6, 14, 17, 18, and 26) for the aggression concept.

#### The Body Investment Scale (BIS)

The body investment scale (BIS) was developed by Orbach and Mikulincer [41] to measure suicidal tendencies and body investment. BIS consists of four subscales (body image feelings & attitudes, comfort in touch, body care, and body protection). A total of 24 items with possible ranging from 24 to 120. Each item uses a 5-point Likert scale (1–5), and seven are reverse-scored. A higher BIS score indicates a higher body investment tendency. The internal consistency of the scale was 0.836 in this study. Five items (3, 5, 13, 17, and 21) were used for the body attitude concept.

#### The Perceived Stress Scale (PSS-10)

The perceived stress scale (PSS-10) was developed by Cohen and Williamson [11]. This scale measures the stress which is perceived as a situation in life. PSS-10 ask about how unpredictable, uncontrollable, and excessive burden is felt in everyday life. A total of 10 items with possible ranging from 0 to 40. Each item uses a 5-point Likert scale (0–4), and four items are reverse-scored. A higher PSS-10 score indicates a higher level of perceived stress. The internal consistency of the scale was 0.762 in this study. Among the ten items, six items (1, 2, 3, 6, 9, and 10) were used for the perceived stress concept.

#### Peer-Victimization Scale & Bullying Behavior Scale (PVS & BBS)

Peer-Victimization Scale & Bullying Behavior Scale (PVS & BBS) was developed by Austin and Joseph [1]. It consists of two subscales (peer-victimization and bullying behavior). Each subscale is consisted of six items and is used by the total score. Each item uses a 4-point Likert scale (1–4), and eight items are reverse-scored. Higher PVS; BBS score indicates having a greater extent and frequency of bully/victim problems. The internal consistency of the scale was 0.924 in this study. We selected five items (1, 3, 4, 5, 6) from the peer-victimization subscale to use for the peer-victimization concept in this study.

#### Family Adaptability and Cohesion Evaluation Scales-IV (FACES-IV)

Family adaptability and cohesion evaluation scale-IV (FACES-IV) was developed by Olson and Gorall [40] and consist of two subscales (adaptability and cohesion). Each subscale is consisted of 10 items and is used by the total score. Each item uses a 5-point Likert scale (0–4). A higher FACES-IV score indicates a good balance between family adaptability and cohesion. The internal consistency of the scale was 0.928 in this study. We used ten items (1, 2, 3, 4, 7, 9, 11, 12, 17, 19) for the family adaptability concept.

**Table 1:** The set of items for conducting LPA (Total number of items = 86)

Type	Factor	Concept	Scale	N
Intrapersonal risk	Negative cognition	Negative thinking	ATQ-N	16
		Brooding	RRS	4
		Body attitude	BIS	5
	Emotional vulnerability	Depression	CES-DC	10
		Depressive rumination	RRS	6
		Aggression	BPAQ-SF	7
	Poor coping skills	Difficulties in emotion regulation	DERS-16	13
		Alexithymia	TAS-20	4
Interpersonal risk	Perceived stress		PSS-10	6
	Peer-victimization		PVS & BBS	5
	Family adaptability		FACES-IV	10

#### The Self-Harm Screening Inventory (SHSI)

The Self-Harm Screening Inventory (SHSI) is a concise and self-administered tool designed to assess self-harm behaviors among adolescents. The SHSI was developed and used in the previous studies [29, 45]. This inventory consists of twenty binary items, where respondents answer with a straightforward "yes" or "no." These items aim to inquire about an individual's involvement in self-harming behaviors within the past year. To ensure clarity regarding the assessment of non-suicidal self-injury (NSSI) among adolescents, the SHSI includes a definition of NSSI within the instructions. NSSI is explicitly defined as deliberate self-harming actions carried out without any intent to cause one's own death. Respondents are instructed to select "yes" if they have engaged in self-harm at least once during their lifetime, and "no" if they have not.

### Procedures

The selection of measurements for this study was guided by their strong psychometric properties, ensuring reliable and valid assessment (see Measures for detailed information). These measures were carefully chosen to capture both intrapersonal and interpersonal risk factors associated with self-harm among adolescents. The decision-making procedure regarding the scale items was informed by a thorough review of existing literature and consultation with psychiatrists and clinical psychologists within the research team. In consultation with these expert, scales that were not statistically and content-wise appropriate were revised or deleted to take account Korean culture. The intrapersonal factor was further divided into three sub-factors: negative cognition, emotional vulnerability, and coping skills. These sub-factors were considered essential in understanding the intrapersonal aspects relevant to self-harm behaviors. Likewise, the interpersonal factor comprised three sub-factors: peer victimization, family adaptability, and perceived stress. These sub-factors aimed to encompass significant elements of interpersonal relationships and external stressors contributing to self-harm behaviors in adolescents. At this stage, the items were initially selected based on the intercorrelations of total scores for each of the 11 psychological scales. Subsequently, any items that did not have respondents who rated them as "Strongly Disagree" (= 1) or "Strongly Agree" (= 5) were further eliminated after examining the response distributions for each item [39]. To finalize the selection of the items, we assessed whether any items exhibited factor loadings below 0.30 or demonstrated high factor loadings across multiple factors, suggesting potential variable complexity (Guadagnoli and Velicer, 1988). To comprehensively assess these factors, a total of 87 items were selected for inclusion in the measures, allowing for the conduct of latent profile analysis (LPA). The final set of items for each sub-factor can be found in Table 1.

### Statistical analysis

The mean score of each factor was considered as continuous indicator variables. Table 2 presents descriptive statistics of each indicator variable. Based on six factors, LPA was conducted to identify latent classes that shows similarity among self-harming behavior factor patterns. All statistical analyses were conducted using Mplus Version 8.4 [33], and the analyses used maximum likelihood parameter estimates with standard errors (MLR). This process used 1,000 random starting values to ensure the validity of each class solution. The number of latent classes was established with a single latent class, and then additional classes were added in sequence, until satisfying an optimal selection criterian. In this study, the optimal number of classes was determined by using Akaike Information Criterion (AIC), Bayesian Information Criterion (BIC), Adjusted BIC (ABIC), the Lo-Mendell-Rubin adjusted Likelihood Ratio Test (LMR-LRT), Bootstrapped Likelihood Ratio Test (BLRT). Lower AIC, BIC and ABIC values indicate a better model fit. The LMR-LRT and BLRT compared a model with *k* classes with a model with *k*—1 classes. If model comparison is statistically significant, it means that a model with *k* classes is better than a model with *k*—1 classes. Whereas a non-significance indicates that a model with *k* classes is not improvement over a model with *k*—1 model. Entropy index is generally useful for providing a summary of classification accuracy. Entropy index varies from 0 to 1, with values closer to 1 indicating less classification errors.

**Table 2:** Descriptive statistics of each indicator variable (sample = 881)

Variables	Min	Max	Range	Mean	SD	Skewness	Kurtosis
CES-DC	0	58	58	18.19	10.37	.79	.66
ATQ-N	29	145	116	53.83	24.04	1.41	1.69
K-RRS	22	86	64	37.64	12.87	4	.88
DERS-16	16	80	64	33.02	14.52	.99	.42
TAS-20	9	42	33	19.40	7.61	.61	-.28
BPAQ-SF	12	57	45	23.46	8.87	1.11	1.01
BIS	20	80	60	58.27	9.70	-.38	-.02
PSS-10	0	24	24	10.87	3.91	.31	.60
PVS & BBS	12	45	33	21.98	7.12	.28	-.69
FACES-IV	0	72	72	47.37	11.85	-.80	1.21

## Results

### The optimal number of classes in the LPA

To identify the optimal number of classes, this study compared models with 1 to 5 classes. Table 3 provides the AIC, BIC, ABIC, LMR-LRT, BLRT and entropy index for comparing these models. In Table 3, the information criteria (IC) decreased sequentially from 1 to 5 class. LMR-LRT and BLRT values for the 2-class, 3-class, and 4- class LPA solutions were significant at *p* = 0.01, whereas the 5-class model was not statistically significant at LMR-LRT index. Thus, the 5-class model was not considered as a best fit model for LPA.

**Table 3:** Information criteria and statistical significance of five models for LPA

Model	AIC	BIC	ABIC	LMR-LRT	BLRT	Entropy
1-class	18,308.223	18,403.845	18,403.845	-	-	-
2-class	14,162.228	14,310.441	14,211.992	<.01	<.01	.953
3-class	12,349.043	12,549.847	12,416.464	<.01	<.01	.928
4-class	11,405.238	11,658.634	11,490.317	<.01	<.01	.941
5-class	10,948.257	11,254.244	11,050.994	.08	<.01	.913

AIC = Akaike Information Criterion, BIC = Bayesian Information Criterion, ABIC = Adjusted BIC, LMR-LRT = the Lo-Mendell-Rubin adjusted Likelihood Ratio Test, BLRT = Bootstrapped Likelihood Ratio Test

Consequently, it is not necessary to test model with above five classes. In comparison of the 3-class with the 4-class model, the improvement of the 4-class model over the 3-class model was negligible (∆ABIC = -757.83). The overall classification accuracy (entropy) for the 3-class model was 0.928, whereas the 4-class model was 0.941. In the 3-class model, the percentage of individuals correctly classified were 96.9% for the first class, 95.9% for the second class, and 98.5% for the third class. For the 4-class model, the percentage of individuals correctly classified were 87.4% for the first class, 85.3% for the second class, 83.6% for the third class, and 80.2% for the fourth class. In result, the 3-class model showed greater parsimony and interpretability than the 4-class model. Thus, the 3-class model was accepted as the final model in this study. The first, second, and third class in the 3-class model consisted of 48% (N = 416), 38% (N = 338), and 14% (N = 127) of the sample, respectively.

### One-way ANOVA analysis for the 3-class model

The one-way ANOVA was conducted to examine the difference among the identified latent classes. In Table 4, the result reported significant differences among the subgroups in terms of negative cognition [F(2,878) = 1386.445, p < 0.001], emotional vulnerability [F(2,878) = 1530.519, p < 0.001], poor coping skills [F(2,878) = 1646.261, p < 0.001], peer-victimization [F(2,878) = 139.569 p < 0.001], family adaptability [F(2,878) = 128.929, p < 0.001] and perceived stress [F(2,878) = 392.689, p < 0.001]. Additionally, the one-way ANOVA was conducted to examine the difference of frequency of self-harm among the identified latent classes. The frequency of self-harm was computed by summing 20 items related self-harming scale. In Table 4, the result reported significant differences among the subgroups in terms of frequency of self-harm [F(2,861) = 153.923, p < 0.001].

**Table 4:** Class differences in six risk factors related to Self-Harming Behavior

	First class mild mean (SD)	Second class moderate mean (SD)	Third class severe mean (SD)	F	Bonferroni
Negative cognition	2.27(0.29)	1.81(0.54)	3.25(0.46)	1386.445***	1 < 2 < 3
Emotional vulnerability	1.51(0.28)	0.91(0.21)	2.46(0.47)	1530.519***	1 < 2 < 3
Poor coping skills	2.30(0.43)	1.39(0.31)	3.65(0.55)	1646.261***	1 < 2 < 3
Peer-victimization	1.99(0.52)	1.56(0.52)	2.40(0.58	139.569***	1 < 2 < 3
Family adaptability	2.55(0.65)	3.04(0.58)	2.10(0.69)	128.929***	1 > 2 > 3
Perceived stress	2.00(0.44)	1.44(0.48)	2.73(0.55)	392.689***	1 < 2 < 3
Frequency of self-harming	0.99(1.14)	1.73(1.97)	5.17(4.79)	153.92***	1 < 2 < 3

^***^p <.001

### Profiling in LPA

**Fig. 1:**
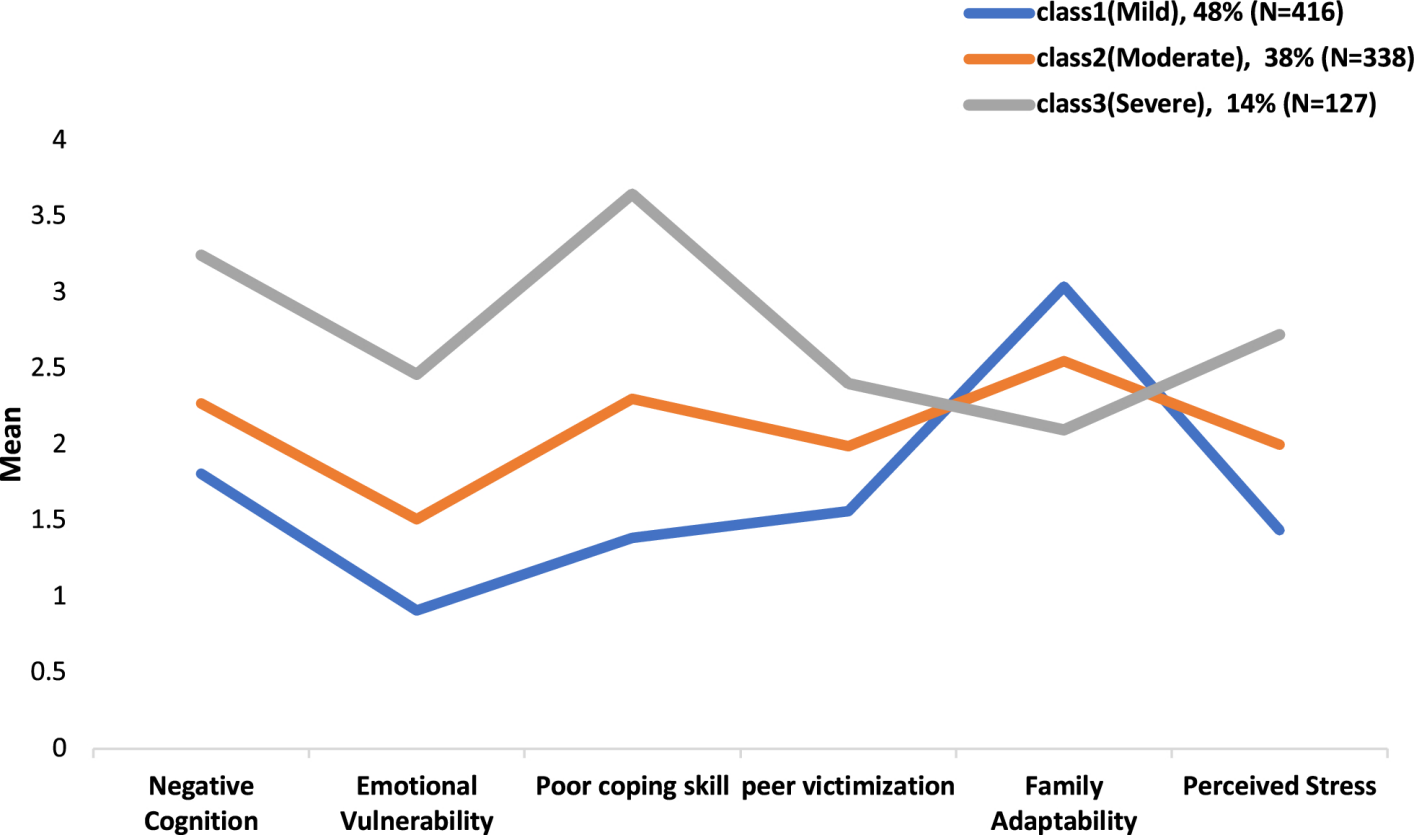
Profiling graph of 3-classes

Based on six factor scores (negative cognition, emotional vulnerability, poor coping skill, peer victimization, family adaptability, perceived stress), three classes are labelled as the “severe” group, “moderate” group, and “mild” group. In Fig. 1, the “severe” group is strongly recommended for treatment intervention programs. In case of “severe” group, the levels of factors that can cause self-harm such as negative cognition, emotional vulnerability, poor coping skill, peer victimization, perceived stress were significantly higher compared to other two groups (“moderate” group and “mild” group), while the level of factors which can hinder self-harm such as family adaptability was very low. On the other hand, the “moderate” group exhibited intrapersonal and interpersonal risk factors levels falling between the severe and the mild groups. Under content specialists, profile data can be used to plan treatment interventions or develop coping strategies for designated latent groups.

## Discussion

Based on Nock’s [35] integrated model of NSSI, the present study aimed to identify subgroups and explore the distinctive features associated with each group among adolescents. The current study utilized latent profile analysis (LPA) to uncover latent classes based on intrapersonal and interpersonal risk factors related to self-harm behaviors as proposed by Nock [35]. Utilizing six factor scores (intrapersonal factors: negative cognition, emotional vulnerability, & poor coping skills; interpersonal factors; peer victimization, family adaptability, & perceived stress), the findings of the LPA revealed three distinct latent classes labelled as the "severe" NSSI group, the "moderate" NSSI group, and the "mild" NSSI group. The follow-up analysis revealed significant differences among the groups not only in intrapersonal and interpersonal factors but also the frequency of NSSI. Consistent with previous research on latent class analysis [13, 14, 21, 22, 54], the “severe” NSSI group exhibited the most pronounced profiles of both intrapersonal and interpersonal problems, whereas the “mild” group displayed opposite patterns (see Fig. 1). Additionally, the “severe” NSSI group reported the highest frequency of NSSI, whereas the “mild” NSSI group reported the lowest frequency of NSSI.

The “severe” NSSI group exhibited the highest levels of negative cognition, emotional vulnerability, poor coping skills, peer victimization, and perceived stress whereas the level of family function was the lowest. These findings are consistent with a previous study in which the severe group, characterized by high NSSI-high suicidality, demonstrated the poorest intrapersonal and interpersonal outcomes, including greater internalizing and externalizing problems and lower social support from both family and friends [13]. In case of “severe” group, the level of factors that can cause self-harm was strongly high compared to other two groups, while the level of factor which can hinder self-harm (e.g. family adaptability) was very weak. On the other hand, the “moderate” group exhibited intrapersonal and interpersonal risk factors levels falling between the severe and the mild groups. However, there is a potential risk of being into a “severe” group depending on the specific conditions. The “mild” group can be defined that they have support from their family and have ability to overcome self-harm behavior compared to the severe and moderate group.

The "severe" group exhibited high levels of negative cognitive patterns, as indicated by their elevated scores on the items related to automatic negative thoughts (i.e., “I’m so disappointed in myself”, “I’m worthless”, “I wish I could just disappear”) brooding (“what am I doing deserve this?”, “Think “ why do I always react this way?”, Think “ why do I have problems other people don’t have?”), and negative body image feelings (e.g., “I hate my body”, “ I feel anger toward my body”). These results are in line with previous studies that negative cognition such as automatic negative thinking and negative information processing biases are significant risk factors of self-harm [7, 17]. As indicated in a theoretical model of self-harm [34], these negative cognitive patterns may exacerbate their emotional distress, making them more susceptible to engaging in self-harm as a maladaptive coping mechanism in response to stressors. For the severe group, emotional vulnerability and poor coping skills manifested as a profound susceptibility to emotional distress, rendering them more prone to engaging in self-harm as a maladaptive coping mechanism. This vulnerability can manifest in various situation, such as academic pressure during exam periods, tumultuous family situations, or social isolation, all of which may exacerbate their emotional vulnerability and lead to self-harm behaviors. During adolescence, accessing healthy and adaptive coping strategies to regulate emotional distress is crucial. However, failure to access these strategies may lead to inward anger, resulting in harsh self-criticism and self-punishment, thereby precipitating NSSI [4, 9, 10, 43]. It’s also important to recognize that emotional vulnerability is not a choice but rather a complex interplay of genetic, psychological, and environmental factors. Understanding this aspect is crucial when developing targeted interventions for individual with self-harm tendencies, as addressing emotional vulnerability can be a key component of treatment strategies aimed at reducing self-harm behaviors.

The "moderate" group displayed intermediate levels of intrapersonal risk factors, positioning them between the severe and mild groups. These individuals exhibited an increased potential for transitioning into the "severe" group under specific conditions. For instance, if they encounter a sudden increase in stressors, such as academic challenges or interpersonal conflicts, they may be more prone to self-harm as a coping mechanism. It is important to closely monitor and provide targeted interventions for this group to prevent the escalation of self-harm. Enhancing their coping skills and addressing any negative cognitive patterns or emotional vulnerabilities may help mitigate their risk. On the other hand, the "mild" group exhibited lower levels of intrapersonal risk factors. These individuals demonstrated a reduced tendency towards negative cognitions and emotional vulnerabilities.

In terms of interpersonal factors, the severe group reported higher levels of peer victimization, indicating a higher likelihood of experiencing bullying or social rejection. These adverse interpersonal experiences may contribute to their self-harm behaviors as a response to the distress and isolation they face. These findings align with prior research emphasizing the role of social support from family as a significant buffering factor in NSSI [24, 32, 47, 49]. In comparison, the "moderate" group demonstrated moderate levels of peer victimization and family adaptability. While they experience some degree of interpersonal challenges, they do not reach the same severity as the “severe” group. Interventions that address both peer-related difficulties and family dynamics can promote better coping mechanisms and a supportive environment for this group. The "mild" group, in contrast, reported lower levels of peer victimization and higher levels of family adaptability. These findings suggest that supportive family environments and positive social relationships may serve as protective factors, buffering against self-harm behaviors in adolescents as the previous study indicated [49].

Overall, these results emphasize the importance of considering both intrapersonal and interpersonal factors when understanding self-harm behaviors among adolescents. Our findings align with previous literature review and research that emphasize the significance of both intrapersonal and interpersonal risk factors in adolescents’ self-harm [13, 17, 34]. By identifying distinct subgroups and their unique profiles, tailored interventions can be developed to address the specific needs of each identified subgroup. For the severe group, characterized by high levels of negative cognition, emotional vulnerability, poor coping skills, peer victimization, low family adaptability, and perceived stress, intensive and comprehensive interventions are warranted. These may include individual therapy focused on cognitive restructuring, emotion regulation skills training, and coping strategies through cognitive-behavioral interventions, mindfulness-based practices, and emotional regulation training [5]. This can help adolescents develop healthier coping strategies, improve problem-solving skills, and enhance emotional resilience. Group therapy sessions can also be beneficial, providing a supportive environment for sharing experiences and learning from peers who have similar struggles [26]. Additionally, involvement of parents and families in family therapy can help strengthen family adaptability, improve communication, and foster a supportive and nurturing environment [15, 52].

Given the potential for transition from the "moderate" group to the "severe" group, regular follow-up and monitoring are essential. Clinicians should maintain ongoing communication with adolescents at risk for self-harm to track their progress, assess any changes in risk factors, and provide necessary support and intervention as needed. Regular check-ins and monitoring can aid in early detection of worsening symptoms and facilitate timely intervention [42]. For the moderate group, interventions should focus on prevention and early intervention to reduce the risk of transitioning to the severe group. Targeted interventions may include psychoeducation on self-harm behaviors, emotion regulation training, and building effective coping skills. School-based interventions such as peer support programs and anti-bullying initiatives can help address peer victimization [46]. Regular monitoring and check-ins with mental health professionals can aid in detecting any escalation of self-harm behaviors and providing timely intervention.

The "mild" group, characterized by family support and resilience, can benefit from interventions aimed at enhancing their existing strengths and protective factors. Psychoeducation and skill-building workshops can further strengthen their coping skills and emotional resilience. Providing information and resources to parents on maintaining open communication, supporting their child's emotional well-being, and identifying signs of distress can be valuable. Encouraging the involvement of these adolescents in extracurricular activities, peer mentoring programs, or community engagement can promote a sense of belonging and provide additional sources of support. Given the prevalence of peer victimization as an interpersonal risk factor, school-based interventions also play a crucial role in preventing self-harm behaviors among adolescents. Schools should implement comprehensive anti-bullying programs and create a safe and supportive school environment. Educational initiatives that promote empathy, emotional regulation, and conflict resolution skills can empower students to address peer victimization effectively and reduce its impact on self-harm behaviors.

In summary, the present study successfully identified three distinct subgroups based on the severity of intrapersonal and interpersonal risk factors. These findings highlight the importance of implementing individual tailored interventions through a module-based approach. Cognitive-behavioral therapy (CBT) techniques targeting negative cognitions could benefit all three groups, while the severe group may require additional longer sessions focusing on emotional regulation skills and mindfulness-based techniques. Addressing emotional vulnerability, an integral aspect of self-harm, should be a core component of intervention. In this sense, Dialectical Behavior Therapy (DBT), specifically designed for individuals struggling with emotional dysregulation and self-harm behaviors, can provide valuable tools for managing intense emotion and reducing self-harm incidents. Ongoing assessment, regular follow-up, and collaboration among mental health professionals, teachers, and parents are crucial for ensuring the effectiveness of early detection and interventions. Furthermore, further research is necessary to evaluate the outcomes and long-term effectiveness of these module-based interventions in reducing self-harm behaviors and promoting overall well-being among adolescents in each subgroup.

While this study contributes valuable insights into the subgroups and factors associated with self-harm behaviors among adolescents, it is important to acknowledge its limitations. Firstly, the study utilized a nationwide online survey, which may have resulted in selection bias. The sample consisted of adolescents and their parents who voluntarily participated, potentially leading to a non-representative sample. Those who did not engage in self-harm behaviors or who were unwilling to disclose their self-harm may not have participated, leading to an underrepresentation of certain subgroups or factors. Secondly, the data collected relied on self-reported measures, which are subject to limitations such as response bias, social desirability bias, and recall bias. Participants may have underreported or misrepresented their self-harm behaviors or other sensitive information. The reliance on self-report measures may introduce measurement error and affect the accuracy of the results. Thirdly, the present study employed a cross-sectional design, which limits the ability to establish causality and infer temporal relationships between the identified factors and self-harm behaviors. Longitudinal studies are necessary to examine the developmental trajectories and changes in the identified subgroups and factors over time. While the study focused on a set of intrapersonal and interpersonal factors, there may be other relevant factors that were not considered. Variables such as trauma history, social support networks, and access to mental health services were not included in the analysis. Future studies could expand the scope of factors examined to provide a more comprehensive understanding of self-harm behaviors among adolescents. Finally, the present study did not consider the versatility and severity of NSSI. A previous study has shown that versatile NSSI, particularly when coupled with depression, is significantly associated with an increased risk of suicide [48]. Therefore, incorporating versatility of NSSI methods may reveal another risk subgroup within the “severe” NSSI group identified in the present study. Acknowledging these limitations helps to contextualize the study's findings and provides directions for future research to address these gaps and strengthen our understanding of self-harm behaviors among adolescents.

Despite these limitations, the present study is the first to identify the latent classes of adolescent’s self-harm in terms of intrapersonal and interpersonal risk factors proposed by Nock’s [35] integrated model of NSSI. The identification of distinct subgroups based on intrapersonal and interpersonal factors allows for the development of targeted treatment interventions. By tailoring treatment interventions, assessing and preventing risks, involving families, implementing school-based interventions, promoting resilience, and ensuring follow-up and monitoring, clinicians can provide comprehensive and effective support for adolescents engaging in self-harm behaviors.

## Supplementary Information

Additional file1

## Data Availability

Data will be available upon reasonable request.
