# Supplementary material for: A latent profile analysis on adolescents' Non-Suicidal Self-Injury related to intrapersonal and interpersonal factors
Source: Child Adolesc Psychiatry Ment Health. 2024 Sep 17;18:120. doi: 10.1186/s13034-024-00801-4 (PMC11409616; doi:10.1186/s13034-024-00801-4)
Supplement: Supplementary file 1 — Additional file1 [file 13034_2024_801_MOESM1_ESM.docx]

**Appendix A**

| Characteristic | Category | Number | Percent(%) |
| --- | --- | --- | --- |
| Gender | Male | 487 | 55.3 |
|  | Female | 394 | 44.7 |
| Grade | 6^th^ Grade | 227 | 25.8 |
|  | 1^st^ Year(Middle School) | 221 | 25.1 |
|  | 2^nd^ Year(Middle School) | 226 | 25.7 |
|  | 3^rd^ Year(Middle School) | 207 | 23.5 |
| Region | Seoul | 188 | 21.3 |
|  | Gyeonggi Region  (Gyeonggi-do, Incheon) | 296 | 33.6 |
|  | Gyeongsang Region  (Gyeongsangnam-do/pukdo, Busan, Daegu, Ulsam) | 205 | 23.3 |
|  | Jeolla Region  (Jeollanam-do/pukdo, Gwanggu, jeju-do) | 95 | 10.8 |
|  | Chungcheong Region  (Chungcheongnam-do/pukdo, Daejeon, Sejong) | 76 | 8.6 |
|  | Gangwon Region | 21 | 2.4 |
